# COVID-19 Pneumonia Presenting with Multiple Nodules Mimicking Metastases: An Atypical Case

**DOI:** 10.5334/jbsr.2681

**Published:** 2022-08-11

**Authors:** Seyma Babaoglu, Necdet Poyraz, Ozlem Sahin

**Affiliations:** 1 Department of Radiology, Meram Faculty of Medicine, Necmettin Erbakan University, Konya, Turkey; 2 Department of Nuclear Medicine, Meram Faculty of Medicine, Necmettin Erbakan University, Konya, Turkey

**Keywords:** COVID-19, computed tomography, multiple, pulmonary nodules, atypical

## Abstract

Coronavirus disease 2019 (COVID-19) is an outbreak causing pneumonia due to severe acute respiratory syndrome coronavirus 2 (SARS-CoV-2) and computed tomography (CT) images are a significant part of the diagnosis of COVID-19 related pneumonia. Typical chest CT findings are bilateral peripheral ground-glass opacities (GGO) with or without consolidation. Although rare, atypical CT findings have been described, no case of COVID-19 causing multiple solid pulmonary nodules has been reported. In this article, atypical CT findings of a 45-year-old female patient with multiple solid pulmonary nodules mimicking metastasis diagnosed with COVID-19 confirmed by reverse transcription polymerase chain reaction (RT-PCR).

**Teaching point:** COVID-19 pneumonia may mimic multiple metastatic nodules radiologically.

## Introduction

COVID-19 is an infectious disease caused by SARS-CoV-2, which emerged in Wuhan, in December 2019. It was declared a global pandemic by the World Health Organization (WHO) on March 11, 2020. As of the end of June 2021, more than 179 million confirmed cases of COVID-19 and 3.9 million related deaths had been reported to WHO. Clinically, fever, cough, fatigue, shortness of breath, joint pain, headache, sore throat, and diarrhea can be seen [1]. Typical chest CT findings by the Radiological Society of North America (RSNA) have been defined as bilateral, peripheral ground-glass opacity (GGO) with or without consolidation, GGO accompanied by interlobular or intralobular septal thickening (crazy-paving pattern), and reverse halo sign in the late period. Atypical findings are isolated lobar or segmental consolidation, discrete small nodules such as tree-in-bud or centrilobular nodules, smooth interlobular septal thickenings accompanied by pleural effusion, and cavitation [2]. Rare cases in which lymphadenopathy, subpleural interstitial thickenings, bullseye sign, solitary pulmonary nodule, and target-shaped combined halo and reverse halo sign have been reported in the literature [3,4,5]. Recognition of rare cases is essential for the correct management of diagnosis and treatment.

To the best of our knowledge, there is no case of COVID-19 presenting with multiple nodules mimicking metastasis in the literature. In this article, we presented a case of COVID-19 with multiple nodules accompanied by GGO, which is confirmed by the RT-PCR test.

## Case Report

**Figure 1:**
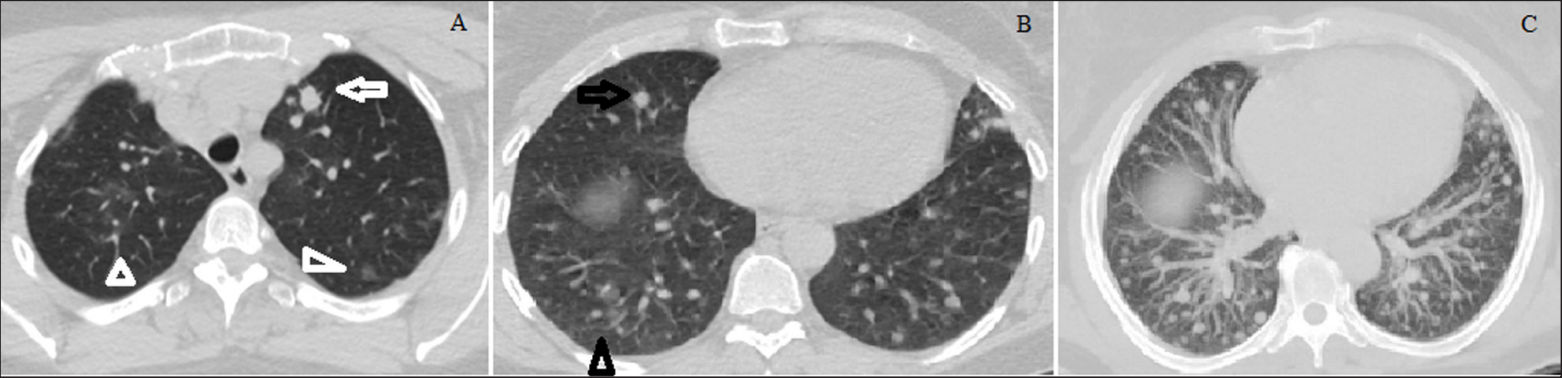
Baseline chest CT shows ground-glass opacities (GGO) (arrowheads) in the bilateral lung parenchyma and multiple solid nodules that the largest one has a diameter of 13 mm (arrow), which is at the anterior segment of the left upper lobe. **(A).** In the lower lobes, many solid nodules with a maximum size of 8 mm on the right upper lobe (black arrow) and GGO in the nodular form (black arrowhead) are observed **(B).** The Maximum Intensity Projection (MIP) image shows bilateral randomly distributed multiple solid nodules.

**Figure 2:**
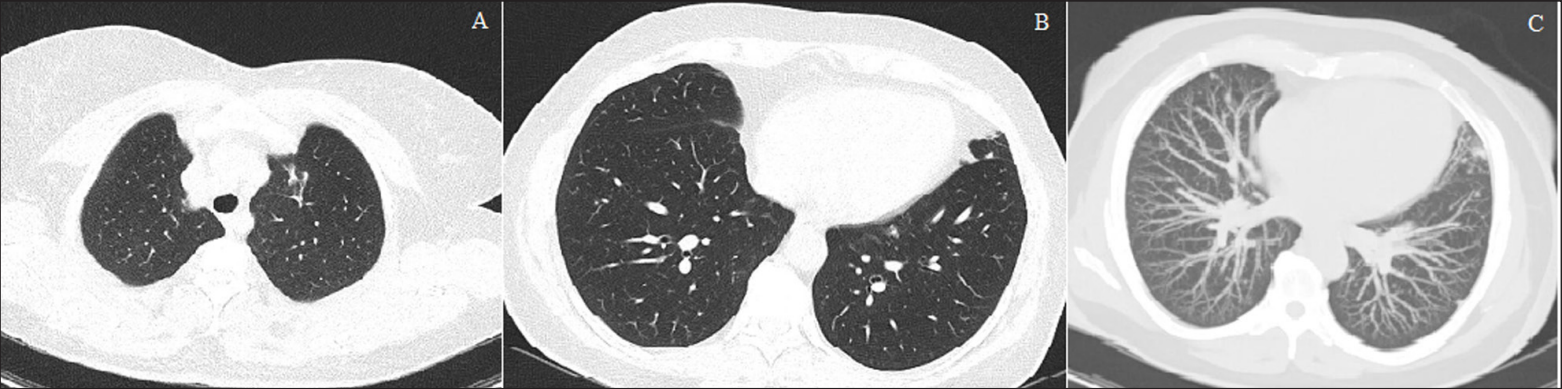
In control chest CT (45 days after disease onset), nodules in the upper lobes and GGO have completely disappeared, and a sequelae band is observed in the place of the nodule in the left anterior segment in Figure 1A. **(A).** Nodules and GGO have regressed almost entirely in the bilateral lower lobes. **(B).** MIP axial CT shows the disappearance of most nodules **(C).**

A non-smoker, the 45-year-old female patient presented to the emergency service with complaints of fever and malaise for ten days and a recent cough on April 26, 2021. Under follow-up of autosomal dominant polycystic kidney disease (kidney function was within normal limits), the patient had no other comorbidities or medication in the medical history. There was no history of exposure. Vital signs other than body temperature (38°C) were within physiological limits. In her laboratory test results, C-reactive protein was 11.7 mg/L, fibrinogen was 764 g/L, and D-dimer was 1.6 mg/L and increased. No abnormality was detected in other parameters. Non-enhanced chest computed tomography (CT) revealed bilateral, diffuse, randomly distributed, sharply defined solid nodules, the largest of which reaches 13 mm in diameter, accompanied by peripheral patchy and nodular GGO (Figure 1). There was no pleural effusion, lymphadenopathy, or cavitation in the nodules. Upon suspicious CT findings for COVID-19, RT-PCR with nasopharyngeal swab was done, resulting in positive; thus, COVID-19 was confirmed. The patient was started on favipiravir (2×1600 mg for two days and 2×600 mg for three days) and paracetamol (4×500 mg) treatment. She was isolated for ten days. Her fever reduced on the third day of treatment, and her cough disappeared on the sixth day. Treatments such as steroid, tocilizumab, and convalescent plasma were not administered to the patient, whose complaints disappeared during the isolation period. No bacterial or fungal growth that could cause multiple nodules was detected in blood and sputum cultures. Also, the other respiratory viral panel was negative. In the laboratory, white blood cell count (WBC), antinuclear antibody, rheumatoid factor, anticitrullinated protein antibodies, and antineutrophilic cytoplasmic antibodies (ANCA) were normal range. Abdominal ultrasonography was routine except for the findings of autosomal dominant polycystic kidney disease. Fluorine 18 (^18^F) – fluorodeoxyglucose (FDG) PET/CT performed (11 days after admission) for primary focus detection showed multiple nodules, some with increased FDG uptake (SUVmax:1.11–6.48) in bilateral lung parenchyma; we observed no other pathological findings. Mammography and breast ultrasonography were normal (BI-RADS 1). Fine needle aspiration biopsy was performed from the nodules (TI-RADS 2-3) detected in the thyroid ultrasonography, and the pathology result was reported as benign. We could not identify the primary focus; therefore, a percutaneous transthoracic biopsy of the pulmonary nodules was planned. In the chest CT (45 days after the baseline CT), it was observed that the GGO disappeared completely; while most of the nodules disappeared, some of them became significantly smaller, and the biopsy was canceled (Figure 2). Diseases that could cause nodules were excluded clinically, with laboratory tests and radiologically. The findings were considered related to COVID-19. After all, control CT was suggested to the patient who had no complaints.

## Discussion

The chest CT has high sensitivity and low specificity in the diagnosis of COVID-19 [6]. Therefore, it is necessary to distinguish between COVID-19 and diseases that cause similar CT findings. Being aware of the rare CT findings in COVID-19 patients leads to early isolation and treatment. Typical CT findings are well known; bilateral, predominantly peripheral GGO with or without consolidation and crazy-paving pattern. Rare findings are bronchial wall thickening, mucoid impaction, nodules (tree-in-bud, centrilobular or solitary nodule), pleural effusion, and lymphadenopathy [2,3]. It has been reported that COVID-19 infection may cause different findings because it is a radiological mimicker [7]. In the literature, there are cases of multiple pulmonary nodules concurrent with RT-PCR positivity in patients. However, in these cases, pulmonary metastasis of primary malignancies are synchronized with COVID-19 [8]. To our knowledge, there has been no case of COVID-19 presenting with multiple solid nodules without primary malignancy or other infectious-inflammatory causes. The differential diagnosis of multiple or diffuse pulmonary nodules is quite broad compared to solitary nodules. Therefore, it is necessary to exclude other diagnoses clinically and with laboratory tests. Distribution, morphological features, and nature of the nodules are also important in the differential diagnosis. Nodules according to secondary pulmonary lobule anatomy show perilymphatic, centrilobular, and random distribution. Differential diagnosis of randomly distributed multiple pulmonary nodules includes hematogenous metastases, tuberculosis, fungal infections, granulomatosis with polyangitis, septic embolism, rheumatoid arthritis, benign metastasizing leiomyoma, and nodular pulmonary amyloidosis. While the differential diagnosis of perilymphatic pulmonary nodules includes sarcoidosis, pneumoconioses (silicosis, berylliosis), lymphangitis carcinomatosis, the differential diagnosis of centrilobular nodules includes hypersensitivity pneumonia, langerhans cell histiocytosis, viral infections, edema, and hemorrhage [9,10]. When the border features are examined, metastasis and miliary infections tend to be more sharply defined, whereas infection, edema, and hemorrhage are poorly defined. Whether nodules are ground glass density or solid also gives an idea of benign and malignant differentiation. While nodules with ground-glass opacities are considered benign entities such as infection, the presence of solid components is related to malignant causes. However, the criteria that will mostly suggest benign nodules are the disappearance of the nodule on follow-up, completed calcification, and no increase in size at 2–3 years of follow-up [9]. In our case, random distribution of the pulmonary nodules, solid and sharply circumscribed are suggested hematogenous metastases and miliary infections. But multiple nodules in our patient were associated with SARS-CoV-2 infection because of the absence of primary malignancy, the exclusion of both miliary infections and other etiological causes as clinical and laboratory findings, and most importantly, the disappearance of the lesions within 1.5 months without any treatment other than antiviral therapy for COVID-19.

## Conclusion

Radiologists should be familiar with the typical findings as well as atypical CT mimickers of COVID-19. We think it is crucial to determine the unusual imaging findings to understand better the pathophysiology and different features of COVID-19 disease. The diagnosis should be confirmed by laboratory tests in patients with clinically suspected COVID-19 presenting with multiple pulmonary nodules.

## References

[1] WHO. Index @ Covid19.Who.Int [Internet]. 2020; 1. Available from: https://covid19.who.int/.

[2] Simpson S, Kay FU, Abbara S, et al. Radiological Society of North America Expert Consensus Document on Reporting Chest CT Findings Related to COVID-19: Endorsed by the Society of Thoracic Radiology, the American College of Radiology, and RSNA. Radiol Cardiothorac Imaging. 2020; 2(2): e200152. DOI: 10.1148/ryct.2020200152 33778571 PMC7233447

[3] Shaghaghi S, Daskareh M, Irannejad M, Shaghaghi M, Kamel IR. Target-shaped combined halo and reversed-halo sign, an atypical chest CT finding in COVID-19. Clin Imaging [Internet]. 2021; 69: 72–4. July 2020. DOI: 10.1016/j.clinimag.2020.06.038 32682246 PMC7329690

[4] Lau JYC, Khoo HW, Hui TCH, Kaw GJL, Tan CH. Atypical chest computed tomography finding of predominant interstitial thickening in a patient with coronavirus disease 2019 (COVID-19) pneumonia. Am J Case Rep. 2020; 21: 1–6. DOI: 10.12659/AJCR.926781 PMC7520135 32952147

[5] McLaren TA, Gruden JF, Green DB. The bullseye sign: A variant of the reverse halo sign in COVID-19 pneumonia. Clin Imaging [Internet]. 2020; 68: 191–6. July. DOI: 10.1016/j.clinimag.2020.07.024 32853842 PMC7834213

[6] Elmokadem AH, Batouty NM, Bayoumi D, et al. Mimickers of novel coronavirus disease 2019 (COVID-19) on chest CT: Spectrum of CT and clinical features. Insights Imaging. 2021; 12(1). DOI: 10.1186/s13244-020-00956-6 PMC7856625 33533965

[7] Duzgun SA, Durhan G, Demirkazik FB, Akpinar MG, Ariyurek OM. COVID-19 pneumonia: The great radiological mimicker. Insights Imaging [Internet]. 2020; 11(1). DOI: 10.1186/s13244-020-00933-z PMC7681181 33226521

[8] Med J, Reports C, Rakhsha A, Fooladi ZM, Jafari A. Simultaneous development of COVID 19 pneumonia and pulmonary metastasis in a known case of chondrosarcoma : A case report. J Med Case Rep [Internet]. 2021; 1–3. DOI: 10.1186/s13256-021-02753-1 33910620 PMC8080480

[9] Karki A, Shah R, Fein A. Multiple pulmonary nodules in malignancy. Curr Opin Pulm Med. 2017; 23(4): 285–9. DOI: 10.1097/MCP.0000000000000393 28463856

[10] Eisenberg RL. Diffuse pulmonary nodules. 2010 May; 354–66. DOI: 10.2214/AJR.10.4345 20410379

